# Recent progress and considerations for AAV gene therapies targeting the central nervous system

**DOI:** 10.1186/s11689-018-9234-0

**Published:** 2018-05-18

**Authors:** Erik Allen Lykken, Charles Shyng, Reginald James Edwards, Alejandra Rozenberg, Steven James Gray

**Affiliations:** 1 Department of Pediatrics, University of Texas Southwestern Medical Center, Dallas, TX 75390 USA; 2 University of North Carolina at Chapel Hill, Gene Therapy Center, Chapel Hill, NC 27599 USA

**Keywords:** Central nervous system, Adeno-associated virus, AAV9, Gene therapy, Clinical trial, Neutralizing antibody, Cellular immunity

## Abstract

**Background:**

Neurodevelopmental disorders, as a class of diseases, have been particularly difficult to treat even when the underlying cause(s), such as genetic alterations, are understood. What treatments do exist are generally not curative and instead seek to improve quality of life for affected individuals. The advent of gene therapy via gene replacement offers the potential for transformative therapies to slow or even stop disease progression for current patients and perhaps minimize or prevent the appearance of symptoms in future patients.

**Main body:**

This review focuses on adeno-associated virus (AAV) gene therapies for diseases of the central nervous system. An overview of advances in AAV vector design for therapy is provided, along with a description of current strategies to develop AAV vectors with tailored tropism. Next, progress towards treatment of neurodegenerative diseases is presented at both the pre-clinical and clinical stages, focusing on a few select diseases to highlight broad categories of therapeutic parameters. Special considerations for more challenging cases are then discussed in addition to the immunological aspects of gene therapy.

**Conclusion:**

With the promising clinical trial results that have been observed for the latest AAV gene therapies and continued pre-clinical successes, the question is no longer whether a therapy can be developed for certain neurodevelopmental disorders, but rather, how quickly.

## Background

The need for long-lasting and transformative therapies for neurodevelopmental disorders cannot be understated. Traditional drug development is made particularly difficult for these disorders due to the blood-brain-barrier (BBB) and off-target effects of drugs affecting neuronal function. Central nervous system (CNS)-directed gene therapy via gene replacement represents a powerful modality to achieve long-term correction of disorders following a single treatment. Multiple vectors exist that can be used for gene therapy, including integrating lentiviral vectors and non-integrating adeno-associated virus (AAV) vectors [1]. While lentiviral vectors offer stable transduction and roughly double the packaging capacity of AAV, in the context of CNS-directed gene therapy, lentiviral vectors have been more amenable to ex vivo treatment approaches and thus far not as amenable as AAV for in vivo gene transfer to widely target the CNS [2, 3]. Even though other viral vectors have shown promise in certain CNS gene therapy applications, this review will focus specifically on the progression towards and beyond the current generation of CNS-directed AAV gene therapeutic strategies. Information on basic AAV biology and vector properties is reviewed elsewhere [1, 2]. Pre-clinical and clinical progress towards the treatment of various neurodevelopmental disorders will be covered to highlight the various challenges and potential therapeutic modalities encountered with AAV gene therapy. Finally, some special considerations for AAV-mediated gene therapy, including potential immune responses, will be discussed.

## Main text

### The evolution of CNS-directed AAV gene therapy

Treatment of neurodevelopmental disorders using AAV vectors represents a tremendous opportunity in the field of gene therapy, though using a modified virus to target the CNS is not without its challenges. The ideal CNS-directed gene therapy will utilize minimally invasive delivery while targeting the appropriate cell type(s) in target tissue(s) to achieve life-long treatment following a single, low dose. The complexity of the CNS, however, poses many obstacles to the ideal AAV gene therapy, including the BBB, invasiveness of delivery, and adequate viral spread from the delivery site [4–8]. Just as for many traditional drugs, the BBB immediately prevents the majority of minimally invasive, peripherally delivered (i.e., intravenous) AAV gene therapies from reaching the CNS. However, the BBB is technically straightforward to bypass by delivering therapy directly to the CNS. A number of pre-clinical studies have demonstrated successful circumvention of the BBB with intracranial gene therapy using intraparenchymal injection [9, 10]. However, this method is highly invasive and, upon translation to higher order mammals, the distribution of AAV particles within the brain is restricted [11, 12]. Overall, intracranial delivery translates to a lower probability of efficacy in larger mammals.

Early CNS AAV studies utilized naturally occurring first (AAV2) and second (AAV5 or AAV8) generation vectors [9, 13–15]. In the last decade, a third-generation vector, AAV9, was determined to have a wide distribution in the brain and spinal cord, targeting both neurons and astrocytes [10, 16]. AAV9, unlike other naturally occurring serotypes, readily crosses the BBB following intravenous injection, thus permitting a minimally invasive treatment modality [16–18]. Further, when injected intracranially or intrathecally, no other AAV serotype has surpassed the distribution of AAV9 [10, 17], permitting overall lower dosing. It has been suggested that AAV9 achieves such wide distribution due at least partially to an ability to undergo axonal transport [10]. AAV9 was additionally exciting because the tropism observed in rodent research models has translated effectively to non-human primates. AAV9 has thus become the gold standard for AAV-mediated gene therapy of the CNS. AAV9 does have limitations in its overall efficiency, however, and there remain questions about whether its cellular tropism (neurons versus glia) translates unaltered between rodents and primates [19]. These questions, along with a relatively high rate of naturally occurring humoral immunity to AAV9 in the general population (~ 47%) [20], have prompted the development of new and hopefully superior AAV capsids.

### Current strategies for the development of next-generation AAV vectors

Like other viruses, AAV tropism is determined by the specific interaction between distinct viral capsid proteins and their cognate cellular receptors (Table 1). Thus, rational design of enhanced AAV capsids requires an understanding of the structural elements in both the capsid and receptor that will permit viral recognition and adhesion. Attempts to use rational design to alter the nature of capsid-receptor binding for gene therapy optimization began in 1999 with a study focused on re-directing AAV2 tropism [21, 22]. This foundational study yielded an AAV2 capsid in which short peptides were inserted into the capsid to disrupt binding to its normal cognate receptor, heparin sulfate proteoglycan, and instead retargeted AAV2 variants to other receptors. Since this study, the structures of many naturally occurring AAV capsids have been solved and their cognate receptors identified [23–42]. With both capsid and receptor structures known, structure-function studies can identify critical binding footprints that mediate tropism, and then capsids can be engineered with the appropriate binding footprint to achieve the desired viral tropism. An example of such an effort is the development of the AAV2i8 capsid [43]. In these studies, the liver-tropic AAV2 capsid binding footprint was swapped for those of the muscle-tropic AAV8, resulting in a novel liver-de-targeted and muscle-targeting capsid. Recent work has further shown that even minor alterations to binding footprints can drastically alter AAV tropism [42, 44, 45]. Rational design therefore offers a powerful tool to manipulate AAV tropism when building upon known binding footprints and receptors. However, the time and resources needed to elucidate capsid structure and receptors for each novel capsid currently limits the impact of rational design approaches and largely confines rational design efforts to a limited, albeit growing, structural toolbox.

**Table 1:** Known cellular receptors for different AAV serotypes

Serotype	Glycan recognition^a^	Coreceptor
AAV1	Neu5Acα2-3GalNAcβ1-4GlcNAc	Unknown
AAV2	6-O- and N-sulfated heparin	Fibroblast [108]/hepatocyte [109] growth factor receptor; laminin receptor [110]; integrin αVβ5 [111] and α5β1 [112]
AAV3	2-O- and N-sulfated heparin	Hepatocyte growth factor receptor [113]; Laminin receptor [110]
AAV4	Galβ1-4GlcNAcβ1-2Manα1-6Manβ1-4GlcNAcβ1-4GlcNAc	Unknown
AAV5	Neu5Acα2-3(6S)Galβ1-4GlcNAc	Platelet-derived growth factor receptor [114]
AAV6	Neu5Acα2-3GalNAcβ1-4GlcNAc; N-sulfated heparin	Epidermal growth factor receptor [115]
AAV7	Unknown	Unknown
AAV8	Unknown	Laminin receptor [110]
AAV9	Galactose [116]	Laminin receptor [110]

^a^All glycan recognitions listed, except for AAV9, are from a study by Mietzsch et al. [117] and are more specific glycans compared to the original publications identifying the primary glycans recognized by each serotype

In contrast to rational design, a directed evolution approach does not require any knowledge of capsid structure or receptor identity. Directed evolution of an AAV capsid starts with the utilization of random mutagenesis, capsid shuffling, and/or random peptide insertions, and follows with a selective pressure to isolate variants with desired properties [7, 46–52]. Random mutagenesis of the capsid DNA, typically by error-prone PCR, introduces amino acid changes to generate a diverse capsid library. DNA shuffling uses random digestion and ligation of a pool of AAV capsid sequences, which, when followed by directed evolution, permits for the potential convergence of unique molecular footprints in one capsid [53–56]. For example, seizure clone 83 is an amalgamation of five AAV serotypes that can cross a seizure-compromised blood-brain-barrier [56]. Relevant capsids are then recovered from the target cell(s) or tissue(s), and then capsids undergo iterative rounds of selection and recovery until a unique population of AAV capsids becomes prominent. Directed evolution has been applied both in vitro and in vivo to generate capsids targeting a variety of different cell types and tissues. Directed evolution thus allows for the discovery of highly specific AAV capsids, relying only on unbiased mutagenesis, selective pressure, and recovery methods. However, from a research standpoint, the power of directed evolution is simultaneously its greatest weakness—a complete lack of understanding of what sequences and structures are critical for a given tropism.

Moving forward, future research studies could feasibly combine rational design and directed evolution methodologies [8, 57]. Indeed, one such study generated a novel AAV2 specific for brain endothelial cells of diseased, but not healthy, animals [58]. Phage display technology was then used to identify unique peptide sequences directed to brain endothelia following intravenous injection, and the peptide was then grafted onto the surface loop region of AAV2. Thus, combining rational design and directed evolution methods can potentially open many avenues for the development of clinically relevant capsids by permitting altered tropism to a desired cell or tissue.

### Pre-clinical and clinical progress

Having a toolbox of vectors with altered tropism is an important step, but to develop an effective therapeutic approach, the cellular and molecular mechanisms driving a disease must be well understood. Whether a disease results from loss of non-cell-autonomous versus cell-autonomous factors, necessary treatment modality, and required expression levels are all critical aspects of gene therapy development and strategy. For example, a loss-of-function defect in a single non-cell-autonomous factor greatly simplifies the development of a successful gene therapy; in this scenario, one need only to introduce a gene product to cells that can secrete that protein, thereby making it available to neighboring cells, which are then able to take up and use the secreted protein. Mucopolysaccharidosis type IIIA (MPS3A, OMIM # 252900) results from the loss of the non-cell-autonomous enzyme heparan N-sulfatase and the accumulation of glycosaminoglycan (GAG). Intravenous administration of AAV9 expressing heparan N-sulfatase in the MPS3A mouse model achieves whole body correction of GAG accumulation and significantly prolongs lifespan [59, 60]. However, the extent of therapeutic benefit critically depends on age at which an animal is treated; mice treated at 3 months of age experienced phenotypic rescue and normalized lifespans, whereas mice treated at progressively later ages failed to normalize lifespan despite improvements in behavior and/or pathology [61].

Disorders driven by loss-of-function in a single cell-autonomous factor provide an additional challenge for gene therapy, because the therapy is only effective if the virus reaches cells specifically deficient in a given gene product. Spinal Muscular Atrophy Type 1 (SMA1, OMIM # 25330) results from the loss of cell-autonomous SMN1 and subsequent motor neuron loss. Intravenous administration of AAV9 expressing SMN1 targeted sufficient numbers of neurons across the CNS and was able to successfully rescue the SMA1 phenotype after neonatal administration in an SMA1 mouse model [62]. Notably, as for MPS3A, treatment at later ages was not effective. Recently, intravenous treatment with an AAV9 vector in an ongoing clinical trial for SMA1 demonstrated dramatic improvements in motor development and survival [63].

It is important to note that the nature of a disease does not always demand a treatment modality with broad coverage of the entire CNS. For example, a current gene therapy strategy for late-onset Parkinson disease (PD, OMIM # 168600) uses administration of L-DOPA, which is converted to dopamine by the enzyme Aromatic L-Amino Acid Decarboxylase (AADC). In PD, the progressive loss of dopaminergic neurons in the substantia nigra results in decreased levels of AADC and a corresponding decrease in L-DOPA efficacy. Direct injection of AAV2 expressing AADC into the putamen led to significantly increased motor performance in patients with PD receiving L-DOPA for the first year post-treatment [64]. However, not all disorders can be successfully treated with direct injection. Indeed, translation of intracranial AAV2 therapies has failed to yield substantial improvements in both Canavan disease (OMIM # 271900), which results from a deficiency in non-cell-autonomous enzyme aspartoacylase, and late-infantile neuronal lipofuscinosis (OMIM # 204500), which results from loss of the non-cell-autonomous enzyme tripeptidyl-peptidase-1 [65, 66]. While identifying a definitive cause for the lack of dramatic improvements in these trials represents a Sisyphean task, it seems likely that a lack of sufficient vector spread, due to the use of AAV2 and an intracranial injection, is at least partially responsible.

Disorders in which gene dosage must be precisely maintained present even greater complications for the development of a successful AAV gene therapy strategy, as is the case for Rett syndrome (RTT, OMIM # 312750). RTT is an X-linked disorder resulting from mutations in the *MECP2* gene, and the mosaic nature of MECP2 expression from the mutated X chromosome results in a cell population with variable cell-autonomous MECP2 expression. While the loss of MECP2 is associated with RTT, overexpression of MECP2 can also cause cell death and an RTT-like syndrome [67]. Thus, AAV gene therapy for RTT must broadly target cells but must only permit moderated MECP2 expression in targeted cells. A recent study led to the development of a vector containing the *MECP2* expression cassette with both a modified endogenous *MECP2* promoter to limit transcription and a 3′ UTR with binding sites for microRNAs known to regulate *MECP2* expression. This novel vector led to enhanced therapeutic efficacy with reduced liver toxicity relative to previous vectors [68, 69]. Ongoing efforts are presently focused on tightening MECP2 expression control and specifically targeting the most critical cell types.

Disorders in which a multiplicity of genes drives the underlying pathophysiology can also pose a significant challenge for the development gene therapy strategies. Several genes have been linked to the onset of amyotrophic lateral sclerosis (ALS, OMIM # 105400), including SOD1, C9ORF72, TARDBP, and FUS, though roughly 80% of cases are of unknown etiology [70]. Further, the molecular mechanisms underlying neuronal death are unknown, although studies have suggested that oxidative stress, deficient neurotrophic factor availability, and chronic inflammation are critical factors. Some studies have focused on treating the toxic gain-of-function in SOD1-associated ALS, an approach that led to delayed onset and lifespan extension [71, 72]. However, therapies directed at this single factor would treat only 2% of all ALS patients [73]. Other studies have focused on AAV delivery of neurotrophic factors that might confer neuroprotection to motoneurons and delay disease onset and progression [74], though whether such therapies will translate to humans remains unclear. ALS thus further highlights the need for a comprehensive understanding of the molecular underpinnings of a disease to adapt AAV gene therapy when the appropriate gene(s) for delivery are not clear.

### Special considerations for successful AAV-mediated gene therapies

#### Achieving proper gene dosage and expression of multiple genes

The precise genetic mechanisms underlying some disorders can create unique obstacles in developing optimal gene therapy approaches for presently untreatable conditions. As highlighted above for RTT, certain gene products are toxic if overexpressed, and so great care must be taken to tightly control therapeutic gene expression in this disease. Control of expression levels can occur across several layers, such as by limiting expression to specific tissues through injection route and by selecting capsids with finely tuned specificities. In the absence of a capsid with the appropriate specificity, cell type-specific promoters may be carefully selected to achieve the appropriate level of transcriptional specificity [75]. Additionally, post-transcriptional controls may need to be engineered into the vector to further tune gene expression, including regulatory sequences in untranslated regions or codon selection to limit translation efficiency.

**Fig. 1:**
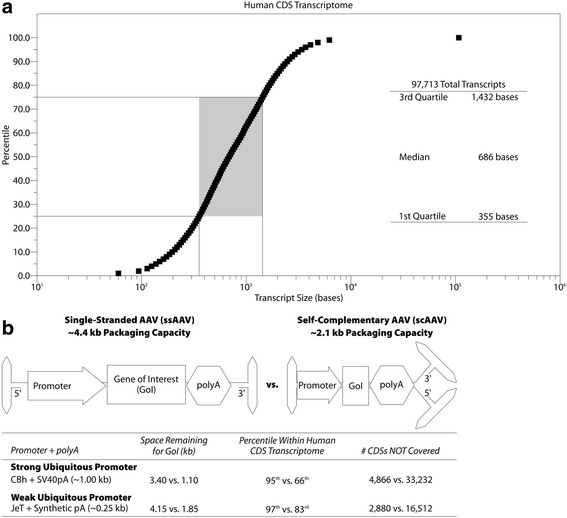
Packaging capacity of AAV. **a** Plot of CDS size in bases versus percentile as determined via analysis of all human CDSs in GENCODE 28. **b** Top panel: cartoons of single-stranded (ss) and self-complementary (sc) AAV with indicated packaging capacities. Bottom panel: calculations of gene of interest size as well as percent of transcriptome covered and number of CDSs not covered at the indicated capacities. The gene/promoter combinations for a relatively strong promoter (CBh) [118] and relatively weak promoter (JeT) [119, 120] are provided as examples to demonstrate relevant gene packaging possibilities

Based on GENCODE 28, > 95% of the 97,713 human coding sequences (CDSs) are under 3.4 kb (Fig. 1a). With a strong ubiquitous promoter, single-stranded rAAV (ssAAV) has sufficient packaging capacity to cover those 92,827 CDSs. The use of self-complementary rAAV (scAAV), which significantly increases transduction efficiency [76], reduces the packaging size to roughly 2.1 kb, permitting coverage of 66% of human CDSs (Fig. 1b). To increase the CDS coverage, often at the cost of promoter strength, different promoters and polyA sequences can be used (Fig. 1b) [75]. However, there still remain disorders in which the gene to be delivered is simply too large for packaging in a single AAV virion. To overcome this limitation, research has focused on packaging the gene of interest across multiple rAAV virions (recently reviewed in [77]). However, this approach requires that a single cell will uptake all the necessary virions—far from a certainty with in vivo administration. Disorders where multiple genes are believed to be responsible (e.g., ALS) represent a similar challenge as packaging multiple genes within the same virion may not be possible due to size constraints. Further, each gene may additionally require distinct regulation to reach critical expression thresholds and/or avoid toxicity. Again, while multiple constructs can be administered simultaneously, there is no guarantee that a single cell will uptake all the required therapeutic genes.

Implicit in the situations described above is the need to achieve an efficient delivery method to the appropriate tissues and cells. Capsid specificity only controls binding to a cell, which necessarily occurs with variable efficiency. AAV must also enter the cell and successfully deliver its therapeutic payload to the nucleus, where the DNA persists outside of the genome, as recombinant AAV is non-integrating. The capsid also controls cell entry and subcellular localization. Thus, generation of highly efficient vectors with appropriate specificity will thus require stringent in vivo screening and selection processes and careful bio-distribution analyses to ensure that the components necessary to permit and enhance entry and trafficking are not lost in directed evolution and rational design approaches.

#### Coping with immune responses

Even a highly efficient and specific vector delivering an ideal transgene means nothing if an immune response prevents AAV from reaching the appropriate cells or, worse, actively destroys cells that receive the therapeutic construct. It is therefore critical when translating AAV9-mediated gene therapy for clinical applications to first determine whether the patient has pre-existing immunity to AAV and to then mitigate the development of potentially damaging immune responses to therapy, particularly when the gene therapy is to be delivered intravenously. While AAV is not presently thought to cause human disease, little effort was initially focused in early pre-clinical studies on the ability of AAV to drive both innate and adaptive immune responses. However, following the observation of an obvious immune response to AAV-based gene therapies during clinical trials [78], studies have begun to evaluate the immune response to AAV-mediated gene therapy in both pre-clinical models [18, 79, 80] and human patients [78, 81].

A pre-existing barrier to AAV-based gene therapy stems from the natural exposure humans receive to AAV, resulting in pre-existing humoral and cellular immunity [20, 82, 83]. Humoral immune responses, derived from antibody-producing B cells, can develop against AAV. At least some anti-AAV antibodies are neutralizing, preventing AAV from infecting cells, and the presence of pre-existing neutralizing antibodies (NAbs) at even low titers (e.g., 1:4) in the serum is sufficient to impede therapeutic efficacy following administration directly into the bloodstream [19, 84]. The low titers required for neutralization raises the prospect of neutralization for therapy delivered directly into the cerebrospinal fluid (CSF) [85]. IgG is the second most abundant protein in the CSF after albumin, (7–12% of total protein) [86]; however, IgG levels in the CSF are 20–1200 times lower than in the serum for healthy children (< 16 years of age) [87] and roughly 300 times lower than in the serum for healthy adults [88]. Thus, pre-existing NAb titers in the serum would need to be reasonably high to expect a clinically meaningful neutralizing response in the CSF, which likely explains why pre-existing NAbs did not inhibit intrathecal therapy in non-human primates [18]. Further, only around 14% of the healthy adult population shows serum antibody recognizing AAV9 above a 1:200 titer [20]. Within the pediatric population (< 18 years of age), the likelihood of high serum NAb titer is even lower, with only around 35% of pediatric samples showing a NAb titer against AAV2 above 1:20 [89], for comparison that goes up to around 90% in healthy adults [20]. Thus, pre-existing NAbs can largely be avoided for first-time gene therapy within the CNS when using direct injection into the brain or spinal cord. In contrast, successful intravenous gene therapy administration will require NAbs to be removed from the serum. However, elimination of NAbs from the serum is not trivial, as it is extraordinarily difficult to eliminate their source, long-lived plasma cells (LLPCs). LLPCs are highly resistant to currently available treatments, including steroids [90] and irradiation [91, 92]. Indeed, elimination of LLPCs has been achieved only through complete immunoablation with antithymocyte globulin [93–95], a treatment that presents substantial risks for patients. The co-injection of empty capsids to act as decoys for NAb binding has also been proposed to circumvent pre-existing NAbs [96]. However, initial pre-clinical studies demonstrated that roughly tenfold higher doses of empty capsid must be used to overcome relatively low (1:1) NAb titers, and even greater relative doses are required to overcome higher NAb titers [96]. Thus, empty capsid administration as an approach to prevent NAb interference is severely limited by the ability to reliably produce sufficient amounts of empty capsid. Further, inclusion of empty capsids only further increases the antigenic load. Ultimately, circumventing pre-existing NAbs to AAV will likely require the development of safe treatments that specifically target LLPCs, though these treatments will then require patients to be vaccinated again post-therapy to reduce vulnerability to common infections.

In addition to extant humoral immunity, pre-existing cellular immunity, particularly that derived from cytotoxic CD8^+^ T cells, represents a threat to successful gene therapy. Indeed, while understandably little work has evaluated T cell responses within the CNS post-therapy, CD8^+^ T cell responses have been observed to limit therapeutic efficacy in gene therapy clinical trials for hemophilia [78, 97]. Pre-existing CD8^+^ T cell immunity poses a greater risk to the patient beyond interfering with therapy, however, as the cytotoxic response can cause significant tissue damage. Activated CD8^+^ T cells release a host of inflammatory mediators to promote immune responses (e.g., CCL3, TNF-α, and INF-γ) as well as cytotoxic molecules enabling direct cell killing (e.g., perforin and granzyme B). Further, T cells are able to establish a local memory pool within the CNS [98, 99]. As for humoral immunity, the rate of pre-existing CD8^+^ T cell immunity to AAV within splenocytes is rather high: ~ 70% in patients 5 years of age and older and ~ 16% in patients under 5 years of age [100]. Further, these pre-existing memory cells are less frequent in the blood than spleen [101] and require multiple rounds of stimulation to observe [100, 101]. These observations highlight the need for detailed study of anti-AAV T cell responses and suggest that pre-existing anti-AAV CD8^+^ T cells are quite likely present in patients. Fortunately, decades of research into transplant rejection and autoimmune diseases have yielded treatments that are able to significantly dampen cellular immune responses, although such treatments may not always be effective prophylactics [102]. Indeed, in some cases, immunsuppressive regimens have led to increases in circulating effector and central memory anti-AAV CD8^+^ T cells [102]. How these therapies may impact long-term immunity requires careful consideration, as long-term immunosuppression poses tremendous and protracted risks, particularly in infants and children. Thorough screening of pre-existing T cell responses (e.g., via multi-parameter flow cytometry) can determine the nature of pre-existing cellular immune responses and permit selection of the most appropriate immunosuppressive regimen, although it remains to be determined whether the screening of T cells in the blood will accurately describe the breadth of pre-existing cellular immunity in a given patient.

Overcoming pre-existing immunity is not trivial, but careful selection of administration routes and immunosuppression regimens following patient screening can go a long way to enable treatment. Once introduced, gene therapy will also induce immune responses to the vector [66, 81, 103, 104] and possibly to the therapeutic transgene. While immune responses to AAV are problematic and may stymie potential re-dosing efforts, responses to the therapeutic transgene pose a much greater risk for patients. Indeed, anti-transgene responses inhibit enzyme replacement therapies in pre-clinical models [105, 106] and may reduce efficacy in humans [107]. While natural features of the therapeutic transgene (e.g., expression level, natural localization of the gene, etc.) will partially determine the overall risk of an immune response to the transgene, efforts to minimize this risk will enhance overall patient safety and therapeutic efficacy. The administration route can control the magnitude and timing of an immune response, with direct treatment of the CNS reducing the risk of severe, systemic immune responses. Constructing selective and efficient vectors to reduce required viral load can also limit the magnitude of immune responses by minimizing inflammation. Immunosuppressive drug regimens can also impede immune activation, although these drugs typically have unintended long-term consequences in infants and children. However, immunosuppression represents a viable option until methods are devised to permit long-term tolerance to the therapeutic transgene while maintaining immunocompetence.

## Conclusions

The progression towards meaningful therapies for neurodevelopmental disorders has greatly accelerated over the past few years. CNS-directed AAV gene therapies, particularly therapies using AAV9, are producing promising pre-clinical results, especially when provided early in the course of disease, and are increasingly translating from the bench into phase I clinical trials. Increasing research to understand the underlying biology of neurodevelopmental disorders will help tremendously to define the relevant cell types and treatment paradigms. When coupled with continued improvements in construct design and the creation of novel AAV variants with specific and enhanced targeting capabilities, there is much hope for the treatment of even complex disorders. As treatments transition into clinical practice, efforts to prevent inhibitory immune responses will be a critical area of focus. Working to understand anti-therapy immunity on the pre-clinical side and careful monitoring during clinical trials will no doubt provide a wealth of insight and help focus the development of safe and highly efficacious therapies.

## Abbreviations

AADC: Aromatic L-Amino Acid Decarboxylase
AAV: Adeno-associated virus
ALS: Amyotrophic lateral sclerosis
BBB: Blood-brain-barrier
CNS: Central nervous system
CSF: Cerebrospinal fluid
GAG: Glycosaminoglycan
ICM: Intra-cisterna magna
ICV: Intracerebroventricular
MPS3A: Mucopolysaccharidosis type IIIA
NAb: Neutralizing antibody
OMIM: Online Mendelian Inheritance in Man
PD: Late-onset Parkinson disease
RTT: Rett syndrome
SMA1: Spinal Muscular Atrophy type 1
